# A retrospective study on the mechanism underlying quick transfer from response to resistance in a repeated recurrent chordoma patient with molecular alterations treated with Palbociclib

**DOI:** 10.1007/s00432-023-05560-x

**Published:** 2024-02-19

**Authors:** Nanzhe Zhong, Dong Yu, Minglei Yang, Xingyi Lu, Qiangzu Zhang, Wei Wei, Jian Jiao, Xinghai Yang, Zhi Zhu, Su Chen, Jianru Xiao

**Affiliations:** 1 Department of Orthopedic Oncology, Shanghai Changzheng Hospital, Naval Medical University, Shanghai, China; 2 Center of Translational Medicine, Naval Medical University, Shanghai, China; 3 State Key Laboratory of Computer Architecture, Institute of Computing Technology, Chinese Academy of Sciences, Beijing, China; 4 Department of Pathology, Changzheng Hospital, Naval Medical University, Shanghai, China

**Keywords:** Chordoma, Palbociclib, Genomic features, Drug resistance

## Abstract

**Purpose:**

There is no approved targeted therapy for chordoma at present. Although several preclinical studies have implied the potential applicability of CDK4/6 inhibitor for this rare tumor, no clinical evidence has been documented so far. The purpose of this study was to elucidate the therapeutic efficacy of CDK4/6 inhibitor for chordoma.

**Methods:**

The next generation sequencing (as for whole-exome sequencing, WES assay) and immunohistochemical (IHC) staining of the chordoma tissue from a patient with an advanced lesion were performed before treatment. Then, the patient was treated with Palbociclib for 4 months until progression occurred in the 5th month. Surgical resection was implemented and the tumor tissue was obtained postoperatively for assessment of molecular alterations.

**Results:**

Molecular features of the tumor before medical treatment suggested applicability of CDK4/6 inhibitor and the patient showed partial response (PR) according to Choi Criteria after 4 months treating with Palbociclib until progression occurred. Then, a drastic molecular alteration of the tumor as represented by emergence of dramatic E2F amplification, which is known to induce CDK4/6 independent cell-cycle entry and progression after treatment, was detected. The findings in this patient demonstrated tumor evolution under drug pressure.

**Conclusion:**

The findings of the present study suggest the feasibility of Palbociclib for the clinical treatment of chordoma, and imply the necessity of combination therapies rather single drug administration due to the quick resistance of the tumor to Palbociclib treatment.

**Supplementary Information:**

The online version contains supplementary material available at 10.1007/s00432-023-05560-x.

## Introduction

Chordoma is a rare malignancy derived from remnants of the notochord, commonly occurring in the axial skeleton. Because of the radio- and chemo-resistance of chordoma, radical resection, sometimes combined with high-dose radiation or the proton radiotherapy remains to be the mainstay of treatment to ensure long-term survival (Stacchiotti and Sommer 2015). However, the local recurrent rate is high due to the anatomical limitation and rarity of adjuvant therapeutic options(Kerekes et al. 2019). Although clinical trials for targeted therapies, such as apatinib (Liu et al. 2020), lapatinib (Stacchiotti et al. 2013), imatinib and everolimus (Stacchiotti et al. 2012, 2018) have been conducted in patients with advanced chordoma, only a small portion of patients possessing the corresponding alterations of molecular targets can benefit. Therefore, elucidation of therapeutic markers harboring in most cases of chordoma will largely facilitate improving current chordoma treatment.

As one of the most prevalent genetic alterations in chordoma, homo- (11.25–30% in chordoma) or heterozygous loss of chromosome 9p21 (about 70% in chordoma) with loci of cyclin-dependent kinase inhibitor 2A/2B (CDKN2A/2B), has been described in several cohorts (Bai et al. 2021; Le et al. 2011; Tarpey et al. 2017). Alterations of CDKN2A/2B, are known to play essential tumor-suppressing roles in a variety of tumors,, making this pathway an attractive therapeutic target (Helsten et al. 2016; Kato et al. 2015). Previous studies suggested that chordoma cells exhibiting a loss of CDKN2A (p16) may trigger universal activation of CDK4/6, implying the potential applicability of CDK4/6 inhibitor in chordoma treatment (Liu et al. 2018) (Passeri et al. 2022). However, no clinical evidence has ever been reported for the effectiveness of CDK inhibitors in the treatment of chordoma.

Here, we present the first case of repeatedly recurrent chordoma in a patient who partially responded to Palbociclib therapy for 4 months and then developed drug resistance within a month. Molecular landscapes of the tumor tissues before and after Palbociclib treatment were obtained by IHC and WES for investigating potential responsive and drug-resistant markers indicating the administration or withdrawal of CDK4/6 inhibitor.

## Case report

**Fig. 1:**
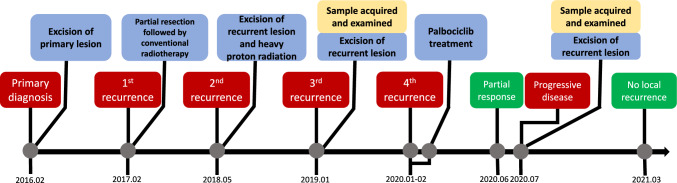
Timeline of patient’s history

**Fig. 2:**
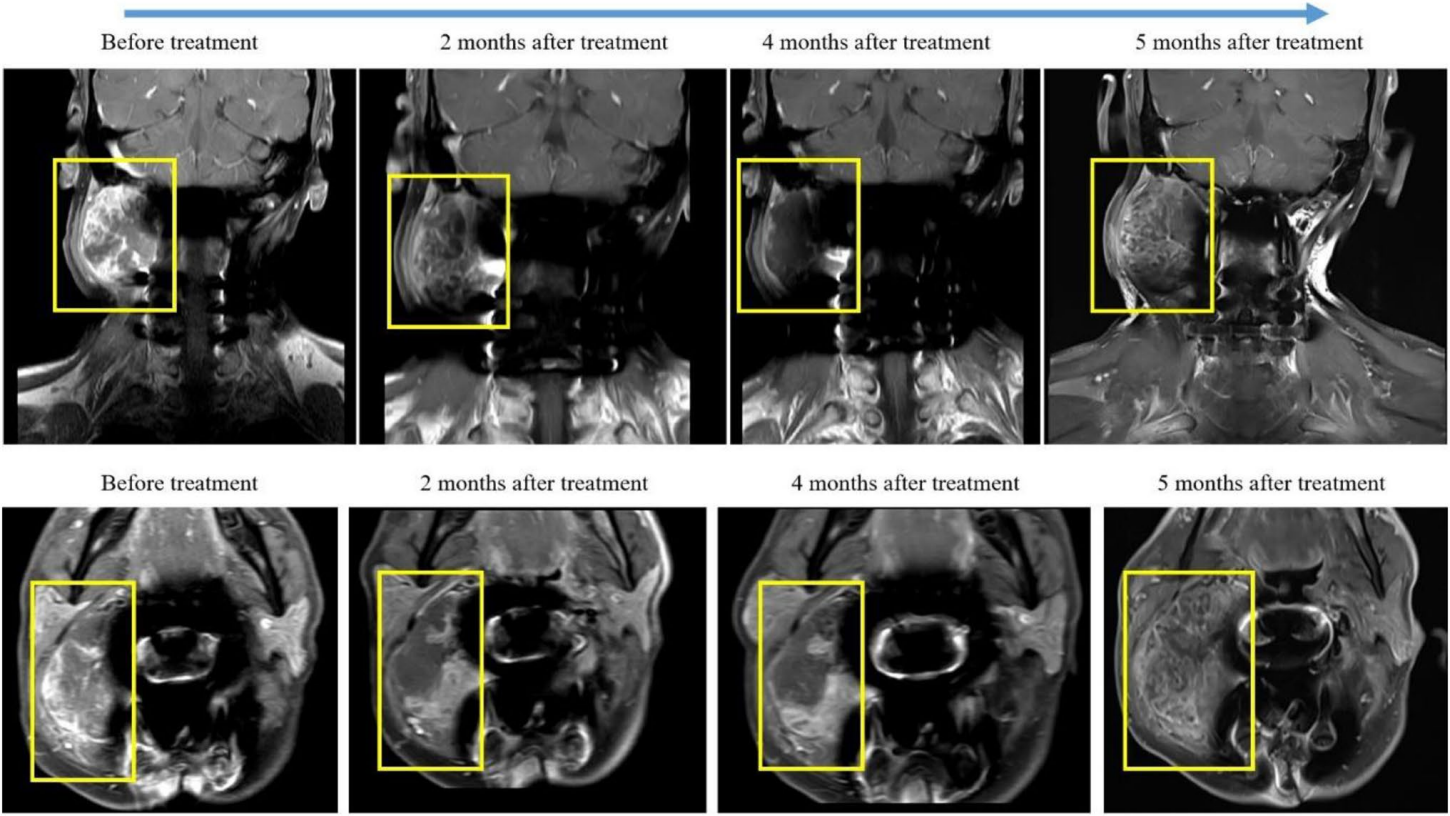
Changes of the tumor size at different time points under Palbociclib treatment

**Fig. 3:**
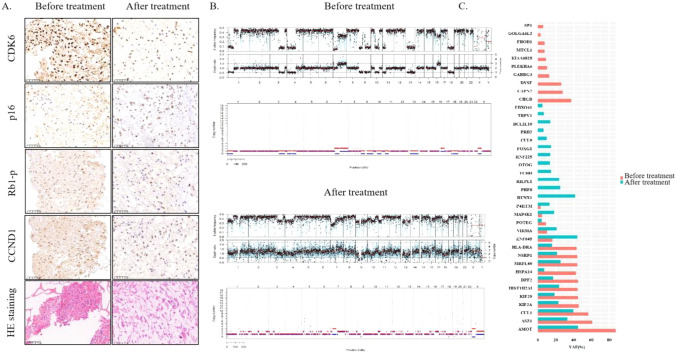
Molecular overview before and after Palbociclib treatment. **A** Protein expression of CDK6, CDKN2A-p16, Rb1-phosphor, and CCND1 of tumor tissues before and after treatment; **B** Somatic copy number alterations (SCNAs) before and after treatment. **A** alleles are depicted in red, **B** alleles in blue; **C** Mutant genes before and after treatment

A 50-year-old man was admitted to our hospital with a history of repeatedly recurrent cervical chordoma. The treatment timeline of this patient is presented in Fig. 1. The lesion involving C2 was first detected when the patient complained of cervical pain and weakness of the lower limbs 7 years ago. The pathological result postoperatively suggested that the lesion was conventional type. Local recurrence occurred one year after partial resection of the tumor, and was treated with conventional radiotherapy. 15 months after the 2nd surgery, the tumor progression led to cervical pain and incomplete paralysis of the extremities. The patient was then referred to our center, where magnetic resonance imaging (MRI) scan revealed a 70 × 47 × 36 mm, well-defined paraspinal pathological mass with bone erosion and remodeling extending to the transverse processes and part of the vertebra bodies, and with a widening of their intervertebral foramina on C2 to C4. A staged posterior-anterior surgery was performed using the 3D printed vertebral body for anterior reconstruction. The pathological result remained to be conventional chordoma. To enhance marginal control, heavy proton therapy was delivered 1 month after surgery. However, local recurrence was detected once again 8 months later during the follow-up period. The tumor was removed and the tumor tissue specimen was obtained. Considering a relative tumor-free surgical margin was achieved in the surgery, adjuvant treatment was not delivered postoperatively. Unfortunately, tumor progression was detected 12 months after operation, when MRI scan showed a 69 × 60 × 73mm pathological mass on the right side (Fig. 2). The patient’s chief complaint was swelling pain of the neck. Tissue obtained from the last surgery were subjected to WES and IHC assays for detection of 719 COSMIC cancer gene alterations (Sondka et al. 2018) (Fig. 3C and Tables 1, 2). Previous studies had observed genetic (Wang et al. 2016) and protein (Cottone et al. 2020) loss of CDKN2A-p16 in different chordoma cohorts, and we also detected a heterozygous deletion that involved the depletion of CDKN2A/2B/2C and gain of CCND2 and CDK6 (Table 2). Meanwhile, IHC staining also showed a loss of CDKN2A-p16, a high expression of CDK6 and positive CCND1 (cyclin D1) in this patient, and nearly half of the cells exhibited phosphorylated Rb1.

**Table 1:** WES analysis for mutant genes before and after treatment

Before treatment					After treatment				
TMB: 0.80; MSI: 3.22					TMB: 1.23; MSI: 4.29				
Gene	Cytoband	Variant	Freq	Type	Gene	Cytoband	Variant	Freq	Type
AMOT	Xq23	exon1 c.836 T > G p.M279R	87.58	Nonsynonymous SNV	AMOT	Xq23	exon1 c.836 T > G p.M279R	46.4516129	Nonsynonymous SNV
ASZ1	7q31.2	exon1 c.14C > T p.A5V	61.31	Nonsynonymous SNV	ASZ1	7q31.2	exon1 c.14C > T p.A5V	33.08823529	Nonsynonymous SNV
CUL1	7q36.1	exon11 c.1298G > A p.S433N	57.96	Nonsynonymous SNV	CUL1	7q36.1	exon11 c.1298G > A p.S433N	38.69463869	Nonsynonymous SNV
KIF2A	5q12.1	exon14 c.1063 T > C p.F355L	46.67	Nonsynonymous SNV	KIF2A	5q12.1	exon14 c.1063 T > C p.F355L	23.72881356	Nonsynonymous SNV
KIF25	6q27	exon4 c.232C > T p.R78C	45.58	Nonsynonymous SNV	KIF25	6q27	exon4 c.232C > T p.R78C	19.45525292	Nonsynonymous SNV
HSPA14	10p13	exon9 c.757G > A p.G253R	45.28	Nonsynonymous SNV	HSPA14	10p13	exon9 c.757G > A p.G253R	6.623931624	Nonsynonymous SNV
DPF2	11q13.1	exon10 c.1011G > T p.Q337H	45.05	Nonsynonymous SNV	DPF2	11q13.1	exon10 c.1011G > T p.Q337H	16.74698795	Nonsynonymous SNV
MRPL40	22q11.21	exon1 c.35C > T p.A12V	44.94	Nonsynonymous SNV	MRPL40	22q11.21	exon1 c.35C > T p.A12V	22.98850575	Nonsynonymous SNV
HIST1H2AI	6p22.1	exon1 c.3G > C p.M1I	44.7	Nonsynonymous SNV	HIST1H2AI	6p22.1	exon1 c.3G > C p.M1I	24.08602151	Nonsynonymous SNV
NSRP1	17q11.2	exon7 c.813G > T p.K271N	44.22	Nonsynonymous SNV	NSRP1	17q11.2	exon7 c.813G > T p.K271N	21.67182663	Nonsynonymous SNV
HLA-DRA	6p21.32	exon4 c.748C > T p.R250C	43.26	Nonsynonymous SNV	HLA-DRA	6p21.32	exon4 c.748C > T p.R250C	15.18987342	Nonsynonymous SNV
ZNF645	Xp22.11	exon1 c.929G > A p.R310H	17.45	Nonsynonymous SNV	ZNF645	Xp22.11	exon1 c.929G > A p.R310H	43.97394137	Nonsynonymous SNV
VIRMA	8q22.1	exon8 c.965A > G p.D322G	10.12	Nonsynonymous SNV	VIRMA	8q22.1	exon8 c.965A > G p.D322G	19.06779661	Nonsynonymous SNV
POTEG	14q11.2	exon6 c.1101A > T p.K367N	8.11	Nonsynonymous SNV	POTEG	14q11.2	exon6 c.1101A > T p.K367N	4.302477184	Nonsynonymous SNV
MAP4K1	19q13.2	exon12 c.826C > T p.Q276X	4.98	Stop gain	MAP4K1	19q13.2	exon12 c.826C > T p.Q276X	18.26401447	Stopgain
P4HTM	3p21.31	exon1 c.137 T > G p.V46G	3.11	Nonsynonymous SNV	P4HTM	3p21.31	exon1 c.137 T > G p.V46G	10.81081081	Nonsynonymous SNV
CHGB	20p12.3	exon4 c.1385G > C p.G462A	37.5	Nonsynonymous SNV	RUNX1	21q22.12	exon9 c.1270 T > G p.S424A	32.14285714	Nonsynonymous SNV
CAPN7	3p25.1	exon12 c.1335_1336del p.E446Sfs*1	26.95	Frameshift deletion	PHF8	Xp11.22	exon13 c.1497delA p.E499Dfs*21	25.30120482	Frameshift deletion
DYSF	2p13.2	exon37 c.3970-2A > G	26.19	NAN	RILPL1	12q24.31	exon7 c.590A > C p.Q197P	16.48351648	Nonsynonymous SNV
GABRG3	15q12	exon3 c.247G > T p.G83C	13.59	Nonsynonymous SNV	TCHH	1q21.3	exon3 c.1595 T > G p.L532W	14.72275335	Nonsynonymous SNV
PLEKHA6	1q32.1	exon12 c.1799G > A p.R600H	10.19	Nonsynonymous SNV	OTOG	11p15.1	exon1 c.161A > C p.N54T	13.44537815	Nonsynonymous SNV
KIAA0825	5q15	exon13 c.2344C > A p.P782T	8.93	Nonsynonymous SNV	RNF225	19q13.43	exon1 c.952 T > G p.W318G	13.43283582	Nonsynonymous SNV
MTCL1	18p11.22	exon12 c.2771G > A p.R924Q	7.16	Nonsynonymous SNV	FOXG1	14q12	exon1 c.289A > C p.K97Q	9.5238	Nonsynonymous SNV
FHOD1	16q22.1	exon21 c.2993G > A p.R998H	7.16	Nonsynonymous SNV	CUL9	6p21.1	exon25 c.4882C > T p.R1628C	8.813559322	Nonsynonymous SNV
SP1	12q13.13	exon6 c.2128A > T p.N710Y	6.03	Nonsynonymous SNV	PRB2	12p13.2	exon3 c.824A > C p.Q275P	6.753246753	Nonsynonymous SNV
					BCL2L10	15q21.2	exon1 c.398A > C p.D133A	6.0729	Nonsynonymous SNV
					TRPV1	17p13.2	exon14 c.1861 T > G p.C621G	4.964539007	Nonsynonymous SNV
					FBXO41	2p13.2	exon1 c.190 T > G p.F64V	4.807692308	Nonsynonymous SNV

*Freq.* Frequency, *TMB* tumor mutational burde, *SNV* single-nucleotide variant, *MSI* microsatellite instability

**Table 2:** WES analysis for somatic copy-number alterations (SCNAs) before and after treatment

Before treatment						After treatment					
Ploidy.mean.cn: 1.85 HRD score: 23 (LOH: 14; Telomeric AI:5; LST: 4)						Ploidy.mean.cn: 2.73 HRD score: 17 (LOH: 0; Telomeric AI:2; LST: 15)					
Gene	Chromosome	Variant	CNt	A	B	Gene	Chromosome	Variant	CNt	A	B
*VHL*	*chr3*	*Amp*	*6*	*5*	*1*	*NOTCH2*	*chr1*	*Gain*	*7*	*5*	*2*
*FANCD2*	*chr3*	*Amp*	*6*	*5*	*1*	*FCGR1A*	*chr1*	*Gain*	*8*	*6*	*2*
*CYP3A4*	*chr7*	*Amp*	*6*	*5*	*1*	*B4GALT3*	*chr1*	*Amp*	*15*	*8*	*7*
						*SDHC*	*chr1*	*Gain*	*7*	*5*	*2*
						*FCGR2A*	*chr1*	*Gain*	*7*	*5*	*2*
						*IKBKE*	*chr1*	*Amp*	*9*	*6*	*3*
						*MAPK14*	*chr6*	*Gain*	*6*	*4*	*2*
						*VEGFB*	*chr11*	*Amp*	*13*	*7*	*6*
						*GSTP1*	*chr11*	*Gain*	*7*	*5*	*2*
						*SMAD3*	*chr15*	*Amp*	*12.25*	*6.75*	*5.5*
						*WNK3*	*chrX*	*Gain*	*6*	*5*	*1*
						*KDM5C*	*chrX*	*Gain*	*6*	*5*	*1*
						*SMC1A*	*chrX*	*Gain*	*6*	*5*	*1*
CDKN2A	chr9	DEL (Het)	1	1	0	CDKN2A	chr9	WT	2	1	1
CDKN2B	chr9	DEL (Het)	1	1	0	CDKN2B	chr9	WT	2	1	1
CDKN2C	chr1	DEL (Het)	1	1	0	CDKN2C	chr1	Gain	3	2	1
CCND1	chr11	WT	2	1	1	CCND1	chr11	Gain	4	2	2
CCND2	chr12	Gain	3	2	1	CCND2	chr12	Gain	4	2	2
CCND3	chr6	WT	2	1	1	CCND3	chr6	Gain	4	2	2
CDK6	chr7	Gain	3	2	1	CDK6	chr7	Gain	4	2	2
CDK4	chr12	WT	2	1	1	CDK4	chr12	WT	2	1	1
E2F1	chr20	WT	**2**	**1**	**1**	E2F1	chr20	Amp	**15**	**8**	**7**
E2F2	chr1	DEL (Het)	1	1	0	E2F2	chr1	WT	2	1	1
E2F3	chr6	WT	2	1	1	E2F3	chr6	Gain	4	2	2
E2F4	chr16	Gain	4	3	1	E2F4	chr16	Gain	4	2	2
E2F5	chr8	LOH	2	2	0	E2F5	chr8	WT	2	1	1
E2F7	chr12	WT	2	1	1	E2F7	chr12	WT	2	1	1
E2F8	chr11	DEL (Het)	1	1	0	E2F8	chr11	WT	2	1	1
RB1	chr13	DEL (Het)	1	1	0	RB1	chr13	Gain	3	2	1

General SCNAs and potential relevant genes related to CDK signal pathway. Note: Ploidy mean cn < 2.7, CNt > 5: Amp; 2 < CNt < 5: Gain; Ploidy mean cn > 2.7, CNt > 9: Amp; 2 < CNt < 9: Gain

The above italic part of the table indicates the major difference of genes altered from before to after treatment. The following part indicates genes involved in CDK signaling pathway. The above bold part of the table highlights the significant of E2F1 amplification after treatment, which might be associated with drug resistance and tumor progression

*CNt* total Copy Number; *A* A allele, *B* B allele, *WT* Wild type, *Amp* amplification, *LOH* loss of heterozygosity, *HRD* homologous recombination deficiency

After multidisciplinary evaluation, Palbociclib was selected to facilitate tumor resection. It was administered orally at a dose of 125 mg daily for 21 days, paused for 7 days, and then continued with the next cycle. In the first few days, the patient reported an obvious relief of the swelling pain of the neck. The follow-up MRI scan performed after 4 months after treatment showed shrinkage of the tumor volume and signs of necrosis. According to the Choi Criteria(van der Veldt et al. 2010), partial response (PR) of the lesion was defined. MRI also showed a decreased rate of enhancement and partial recession of tumor infiltration compared to the pre-treatment MRI (Fig. 2). Blood routine, liver/kidney function, and blood coagulation function were examined biweekly, showing no significant adverse event. However, the patient reported a relapse of the cervical pain during the fifth cycle of treatment. MRI was performed immediately, showing re-progression of the lesion causing compression of the spinal cord (Fig. 2). Hence, surgery was inevitable, and the surgically resected tumor specimen was sent for second WES and IHC assays.

After Palbociclib treatment until re-progression occurred, a proportion of negative CDK6 cells appeared for IHC staining (Fig. 3A), but more gains of CDK6 (CNt from 3 to 4) and CCND1 (CNt from 2 to 4) was observed at genetic level (Table 2). CDKN2A-p16 protein became positive which is consistent with genetic alteration. No significant difference was observed for phosphorylated Rb1 before and after treatment. WES assay showed less chromosomal deletions (including disappearance of chromosome 9p) and more gains and amplifications after treatment when comparing the genetic profiles (Fig. 3B). Besides, some mutations disappeared and others emerged, and the frequencies of the mutant genes shared by both tissues from before and after treatment were significantly reduced after treatment (Fig. 3C; Table 1). Notably, the copy number of E2F1 dramatically increased from the wild type (CNt 2; A allele 1, B allele 1) to amplification (CNt 15; A allele 8, B allele 7) which may imply drug resistance (Huang et al. 2022) (Table 2). Routine postoperative follow-up observation showed no local recurrence 6 months thereafter.

## Discussion

In this study, we firstly reported the response and development of drug resistance to Palbociclib applied in a patient with advanced chordoma. As the tumor tissue specimens were obtained both before and after treatment, molecular portraits reflecting the tumor evolutionary process under medical pressure or the spatial heterogeneity of the tumor mass were captured in this case.

Although no specific parameter correlative to the efficacy of CDK4/6 inhibitors has been reported, amplification of CDK4/6, CCND1, D2, and/or D3, and alterations in CDKN2A/B are suggested as putative markers to predict the response from CDK4/6 inhibitors (Kato et al. 2021). It is reported that co-deletion of CDKN2A and CDKN2C confer Palbociclib-sensitivity in glioblastoma (Wiedemeyer et al. 2010) and soft tissue sarcoma, demonstrating that amplification of CDK4 benefits from the CDK4/6 inhibitor (Mangat et al. 2018). Broto et al. have also assessed the role of Palpociclib in sarcomas overexpressing CDK4 but not CDKN2A and achieved promising results excluding liposarcoma (Martin-Broto et al. 2023). In our case, the tumor specimen presented heterozygous deletions of CDKN2A, CDKN2B and CDKN2C (Table 2), and gains of CDK6 and CCND2. CDKN2A-p16 protein was not detected,accompanied with the positive expression of CDK6 and CCND1 (Fig. 3A). Thus, the CDK4/6 inhibitor Palbociclib was administered to the patient. After 4-cycle treatment, PR of the lesion was defined according to the Choi Criteria. However, the tumor continued progressing thereafter. The relatively short duration of treatment response in our case might be attributed to several reasons. Firstly, the WES assay was not performed right after Palbociclib treatment after the third surgery. The delay of adjuvant targeted therapy might impair the efficacy of treatment. Secondly, the tissue sample analyzed by WES assay might not represent the whole tumor mass due to the spatial heterogeneity, which may result in inaccurate assessment of the Palbociclib sensitivity. In addition, the current biomarkers analyzed might not be precise enough for the prediction of Palbociclib efficacy.

The results of WES assay and IHC staining of the tissues obtained from the third and the fourth surgery were compared to further explore inherent mechanism of alteration in drug sensitivity. Although the protein expression of CDK6 slightly decreased after treatment, the former heterozygous deletion of CDKN2A/2B/2C vanished, and CCND1/2/3 and CDK6 obtained more gains than before as shown by WES assay (Table 2). The divergent genetic landscapes suggest a dramatic change in tumor composition. Since both tumor tissues were taken from the same location, the possibility of spatial heterogeneity could be ruled out. Considering the alteration of sensitivity to the drug, the genetic discrepancy might be the result of tumor evolution under drug pressure.

It is worthy to mention that E2F1 amplification emerged after drug resistance (Table 2), which is considered to be associated with the resistance of CDK4/6 inhibitors due to the compensation of CDK4/6 independent CyclinE-CDK2 cell-cycle entry and progression (Álvarez-Fernández and Malumbres 2020). Previous studies suggest that CDK2 ablation may rescue the sensitivity of resistant cells to CDK4/6 inhibitors in vitro (Gong et al. 2017; Harbour et al. 1999). Our results also suggest that the combined administration of additional drugs such as specific CDK2 inhibitor may provide better outcomes compared with the use of the CDK4/6 inhibitor alone (Kato et al. 2021). Additionally, previous studies suggested that CDK4/6 inhibitors could augment response to immune-checkpoint therapy (Álvarez-Fernández and Malumbres 2020; Fassl et al. 2022). In our case, we observed an increase of tumor mutational burden (TMB) (from 0.80 to 1.23 mutations/Mb), which is widely admitted as a biomarker for response to immunotherapy across multiple cancer types (Samstein et al. 2019).

In summary, our data provide the first clinical evidence and comprehensive genomic profiling including the potential response, and assumed drug resistance markers during application of the CDK4/6 inhibitor in a chordoma patient. The tumor evolution detected in this case demonstrated that, chordoma patients whose tumors harbor potentially sensitizing alterations to G1/S cell-cycle signaling pathway may benefit from CDK4/6 inhibitor, and also implies necessity of combination therapies because of the possibility of drug-resistance generated by single drug administration. Moreover, this study also highlighted the role of next generation of sequencing (NGS) analysis in daily clinical practice for tumors with less mutations (Racanelli et al. 2020). However, considering the limited case number and retrospective nature of this report, the evidence level of this result is relatively low. Besides, as there is a paucity of feasible biomarkers for the evaluation of CDK4/6 inhibitor sensitivity in chordoma (Passeri et al. 2022), another limitation of this research includes that the criteria for application of Palbociclib in the current case might be controversial. Hence, the precise prediction of Palbociclib sensitivity might be undermined, and the spatial heterogeneity of the tumor cannot be completely ruled out, which possibly lead to the reduced duration of treatment response. Indeed, larger prospective trials on Palbociclib treatment and identification of drug-targeted biomarkers in chordoma are required for an unmet need to expand the limited therapeutic options of chordoma.

## Supplementary Information

Below is the link to the electronic supplementary material.

Supplementary file1 (DOCX 25 KB)

## Data Availability

The data are available from the corresponding author on reasonable request. The sequencing data are deposited in the Genome Sequence Archive (GSA) for human under accession number PRJCA017336 (https://ngdc.cncb.ac.cn/gsa-human).
