# Supplementary material for: A retrospective study on the mechanism underlying quick transfer from response to resistance in a repeated recurrent chordoma patient with molecular alterations treated with Palbociclib
Source: J Cancer Res Clin Oncol. 2024 Feb 19;150(2):95. doi: 10.1007/s00432-023-05560-x (PMC10874909; doi:10.1007/s00432-023-05560-x)
Supplement: Supplementary file 1 — Supplementary file1 (DOCX 25 KB) [file 432_2023_5560_MOESM1_ESM.docx]

**Supplementary file**

**Methods:**

**Redacted protocol of the clinical trail:**

Phase 2 Multicenter Trial of Palbociclib in Advanced Chordoma

Inclusion Criteria:

1. Confirmed diagnosis of chordoma (with metastatic lesion or not candidates for surgery or radiotherapy).
2. Patients must have received standard treatments for at least one line (surgery).
3. ECOG 0-1 at the time of inclusion.
4. Disease progression according to RECIST 1.1 or Choi Criteria, within the year prior to inclusion, to previous treatment (surgery or radiotherapy).
5. Patients must have the following lab results: Absolute neutrophil count ≥ 1,500/mm^3^ (1.5 x 10^9^/L); Platelets ≥ 100,000/mm^3^ (100 x 10^9^/L); Hemoglobin ≥ 9 g/dL (90 g/L);
6. The patients must have signed the written consent to participate in the clinical study, and to provide at least 15 tumor slices in paraffin for the molecular analysis.
7. Age between 18 and 85 years (both ages included).
8. At least one molecular feature included: (1) Mutations or somatic copy number alterations of CDKN2A/2B; (2) Expression positive of CDK4/6; (3) Mutations or somatic copy number alteration of genes implicated in cell cycle signaling pathway; (4) Expression negative of CDKN2A/2B.
9. Measurable disease according to RECIST 1.1 criteria or Choi Criteria.

Outcome Measures:

The endpoints were objective response rate (ORR), progression-free survival (PFS) rate and time to progression (TTP). Tumor volume before and after treatment was assessed by enhanced Magnetic Resonance Imaging (MRI) or enhanced Computed Tomography (CT) imaging.

1. Efficacy measured through the progression free survival (PFS) rate at 6 months, evaluated with RECIST 1.1 criteria. The evaluation criteria will be based on the identification of target lesions in baseline and their follow-up until tumor progression.
2. Efficacy measured through response according to Choi criteria measured through response according to Choi criteria: [ Time Frame: 6 months ]. Efficacy measured through response according to Choi criteria. The evaluation criteria will be based on the identification of target lesions in baseline and their follow-up until tumor progression.
3. Efficacy measured through median PFS [ Time Frame: 6 months ]

Rules for dose and dose modification:

Palbociclib will be administered orally at a dose of 125 mg once a day for 21 consecutive days followed by 7 rest days to comprise a complete cycle of 28 days.

Dose modification of Palbociclib: through the evaluation of adverse events (type, incidence, severity, timing of appearance) observed in physical explorations and laboratory tests. Toxicity will be assessed using NCI-CTCAE 4.0. When grade III side effect occurs, withdraw medication until ≦II side effect and then reduce dose to 100 mg/75mg once a day. When grade III side effect with neutropenia, temperature≧38.5℃ and infection or grade IV side effect occurs, withdraw medication immediately until recovered to ≦II side effect.

Reasons for cessation of trial therapy:

Treatment will continue until disease progression, development of unacceptable toxicity, non-compliance, withdrawal of consent by the patient or investigator decision.

The statistical tests:

T-test, chi-square test, and non-parametric test.

The age, sex, involved spinal vertebral bodies, height, weight, and lesion type of the patients were statistically described and tested.

Tumor growth was assessed by imaging data, and patients' long-term prognosis was statistically analyzed and tested using survival analysis.

**Patient：**

The patient had provided written informed consent for the use of his clinical data for research purposes and for publication of this case report. The study protocol was approved by the medical ethics committee (MEC) of Shanghai Changzheng Hospital MEC-Project Number: 2021SL040. The Declaration of Helsinki was respected.

**Histopathology and IHCs**

Immunohistochemistry (IHC) was performed on formalin-fixed, paraffin-embedded (FFPE) tissue sections on the Ventana Ultra XT automated staining System (Ventana Medical Systems, Oro Valley, AZ, USA) using Ventana reagents, according to the manufacturer´s protocol. Tissue sections were stained with an antibody against CDK6 (CQA1886, 1:200, Cohesion, LONDON, UK), Cyclin D1 (CQP677, 1:200, Cohesion, LONDON, UK), p16 (CPQ1210, 1:100, Cohesion, LONDON, UK), phosphor-RB1 (CPA5868, 1:100, Cohesion, LONDON, UK), Brachyury (ab209665, 1:800, Abcam, UK). Primary antibody detection was performed using the OptiView DAB IHC detection kit (Ventana).

**Next-generation sequencing**

Tumor genomic DNA was extracted from the fresh-frozen tissue, and normal-germline DNA was derived from the blood sample following the manufacturer’s protocol (TIANGEN, DP304-03). Exome sequences were selected for using xGen hybridization capture of DNA libraries (IDT) for NGS target enrichment. Whole exome sequencing was performed using the Illumina X-Ten platform. After excluding reads containing adapter sequences and low-quality sequences, the clean data were mapped to the reference human genome (ensemble GRCh37) according to the GATK Best Practice, which is involved in the software Burrows-Wheeler Aligner (VN:0.7,8-r455), SAMtools (v1.8), Picard (v2.18.20) and GATK (v3.8-1-0). BAM-matcher was used to verify whether two BAM files were generated from the same patient.

Further analysis involved the identification of somatic and germline mutations, tumor mutational burden (TMB), somatic copy number alternations (SCNA), and other genomic feature analysis. Mutect 1.4 was used to detect somatic SNVs, and Strelka was used for indel calling. GATK HaplotypeCaller and Strelka2 were used to call germline single nucleotide variants (SNVs) and INDELs with the default settings on the samples. And allelic-specific SCNA analysis was performed using the R package sequenza (version 2.1.2). An arm-level SCNA was defined if the SCNA event covered 90% of the p or q arm of any chromosome. The chromosome-level SCNA was defined if both p and q arm presented the same copy number variation values. The HRD score was determined as a simple sum of the three factors (telomeric allelic imbalance - TAI, large-scale state transitions - LST and loss of heterozygosity - LOH). The Sequenza outputs of each sample were processed by the scarHRD package [8] with a default parameter as previously described.

**Data availability:**

Sequencing data are deposited in the Genome Sequence Archive (GSA) for human under accession number PRJCA017336 (https://ngdc.cncb.ac.cn/gsa-human).
